# Acupressure and Qigong in post-infectious fatigue with impaired physical function: a randomized clinical trial

**DOI:** 10.3389/fmed.2026.1872035

**Published:** 2026-08-10

**Authors:** Joanna Dietzel, Theresa Bauer, Tatjana Tissen-Diabaté, Miriam Ortiz, Julia Siewert, Judith Bellmann-Strobl, Ute Engelhardt, Stephanie Roll, Weronika Grabowska, Carmen Scheibenbogen, Stefan N. Willich, Friedemann Paul, Benno Brinkhaus

**Affiliations:** 1 Charité – Universitätsmedizin Berlin, Corporate member of Freie Universität Berlin and Humboldt-Universität zu Berlin, Institute of Social Medicine, Epidemiology and Health Economics, Berlin, Germany; 2 Charité – Universitätsmedizin Berlin, Corporate member of Freie Universität Berlin and Humboldt-Universität zu Berlin, Institute of Medical Immunology, Berlin, Germany; 3 Charité – Universitätsmedizin Berlin, corporate member of Freie Universität Berlin and Humboldt-Universität zu Berlin, in cooperation with Max Delbrück Center for Molecular Medicine in the Helmholtz Association, Experimental and Clinical Research Center, Berlin, Germany; 4 International Society for Chinese Medicine (SMS), Munich, Germany; 5 Rostock University Medical Center, Rostock, Institute for Biostatistics and Informatics in Medicine and Ageing Research, Rostock, Germany; 6 Charité – Universitätsmedizin Berlin, Corporate member of Freie Universität Berlin and Humboldt-Universität zu Berlin, NeuroCure Clinical Research Center, Berlin, Germany

**Keywords:** acupressure, long COVID, post COVID-19 syndrome, post-infectious fatigue, Qigong, randomized controlled trial, SARS-CoV2

## Abstract

**Background:**

Post-COVID Syndrome (PCS) is characterized by a variety of symptoms including fatigue and impaired physical function. The aim of the ACUQiG-study was to investigate the therapeutic effects and safety of a self-care program combining acupressure and Qigong on physical function in PCS patients with post-infectious fatigue.

**Methods:**

In this two-arm, open-label randomized controlled study, patients with confirmed SARS-CoV-2 infection and persisting fatigue and ≥3 other typical PCS symptoms >12 weeks were enrolled. Patients were randomized to an intervention group receiving an 8-week daily self-applied acupressure regimen performed at home plus twice-weekly online Qigong sessions, or to a control group receiving no study intervention in the first 16 weeks. Both groups received written self-care advice on complementary medicine treatments and continued routine care. The primary outcome was physical function at 8 weeks measured via the SF-36 Physical Functioning Subscale (SF-36 PFS). Secondary outcomes included fatigue measured by the Chalder Fatigue Scale (CFS), responder analysis, and safety outcomes.

**Results:**

A total of 235 participants (85.1% women; mean age 42.1 years) were randomized (*n* = 118 intervention, *n* = 117 controls). At week 8, the adjusted between-group difference in SF-36 PFS was 4.2 points (95% CI −0.9 to 9.4; *p* = 0.109) with a mean of 55.4 (51.8–59.1) in the intervention group vs. 51.2 (47.6–54.9) in the control group. Responder analysis (≥10-point improvement) suggested a favorable trend for the intervention group (58.9% vs. 46.3%; odds ratio 1.69 (95% CI, 0.98 to 2.92, *p* = 0.058). The intervention group showed slightly greater improvements in Chalder Fatigue Scale scores [mean at week 8, 21.4 (95% CI 20.2–22.6) vs. 23.2 (95% CI 22.0–24.3), *p* = 0.040] compared to the control group. Overall, the self-applied study intervention was safe, but aggravation of preexisting symptoms occurred.

**Conclusion:**

Both groups improved over time; the addition of acupressure and Qigong to a self-care advice leaflet did not provide a statistically significant or clinically relevant improvement in physical function compared to the leaflet alone. Further high-quality clinical trials are needed to investigate the effects of multimodal self-care programs for managing PCS-related fatigue and physical function.

**Clinical trial registration:**

Clinicaltrials.gov identifier: NCT05289154, https://clinicaltrials.gov/ct2/show/NCT05289154. First registered 21.03.2022.

## 1 Introduction

Following the start of the SARS-CoV2-pandemic in 2019, a high number of infected patients continue to suffer from chronic fatigue, exertional intolerance, and various other symptoms, including pain, cardiovascular symptoms such as tachycardia or postural tachycardia syndrome, and neurological symptoms such as neuropathies or mild cognitive impairment (brain fog). These sequelae are independent of the severity of the infection (1, 2). The post-COVID-19 syndrome (PCS), as defined by the WHO (12 weeks persistency, include fatigue, shortness of breath, and cognitive dysfunction and symptoms have no other explanation) (3) has affected more than 10% of SARS-CoV2-infected, leading to a high number of patients with chronic course and a subset incapable to work, do household chores or take part in social activities, creating an enormous socio-economic impact (4–6). There are many similarities between PCS and Myalgic Encephalomyelitis/Chronic Fatigue Syndrome (ME/CFS), which frequently develops post-infection, and a subset of patients with PCS indeed fulfil the Canadian Consensus criteria for ME/CFS (2, 4).

The impact of PCS on immune, neurological, and cognitive systems, vascular issues and organ damage, dysautonomia, and respiratory and gastrointestinal pathologies has been described extensively (5, 6). Treatment options for PCS remain limited, primarily due to insufficient understanding of its underlying pathophysiological mechanisms and paucity of development of targeted therapies. Multiple potential contributors have been identified (5, 7), including endothelial dysfunction (8), autoimmune responses, persistent viral reservoirs or ongoing inflammation (9, 10), and direct tissue damage (6).

So far, there are no effective pharmaceutical or non-pharmaceutical treatments available for PCS, and the complexity of the condition necessitates a comprehensive, multimodal therapeutic approach. Therefore, we aimed to investigate a typical non-pharmacological combination of treatments from Chinese medicine: acupuncture point stimulation and Qigong exercise, which can be performed by patients at home. Previous clinical trials have demonstrated positive evidence for the use of acupuncture and Qigong in addressing one of the primary symptoms of PCS: chronic fatigue (11–13). Acupressure, a needle-free technique of Chinese Medicine (CM), involves stimulating defined acupuncture points through pressure, such as with a fingertip, and can be self-applied by patients. This method has been evaluated and shown to be effective for other forms of fatigue and various conditions (14–17). Qigong, a preventive and therapeutic practice of CM, combines physical exercises, relaxation, deep breathing, and visualization. Clinical trials suggest that Qigong may benefit patients with ME/CFS, particularly in alleviating fatigue (12, 18). Despite this evidence, studies specifically investigating the effects of a combination of acupressure and Qigong in PCS patients are lacking.

The study aim was to investigate the therapeutic effects and safety of self-applied acupressure and online Qigong in PCS patients within the “Self-applied ACUpressure and Online QiGong for Chronic Fatigue Following COVID-19” (ACUQiG)-study. The primary hypothesis was to test whether the combination of acupressure and qigong improves physical function in PCS patients with fatigue compared to the controls, and secondarily if it improves measures of fatigue, sleep, and quality of life.

## 2 Methods

### 2.1 Design and setting

**Figure 1:**
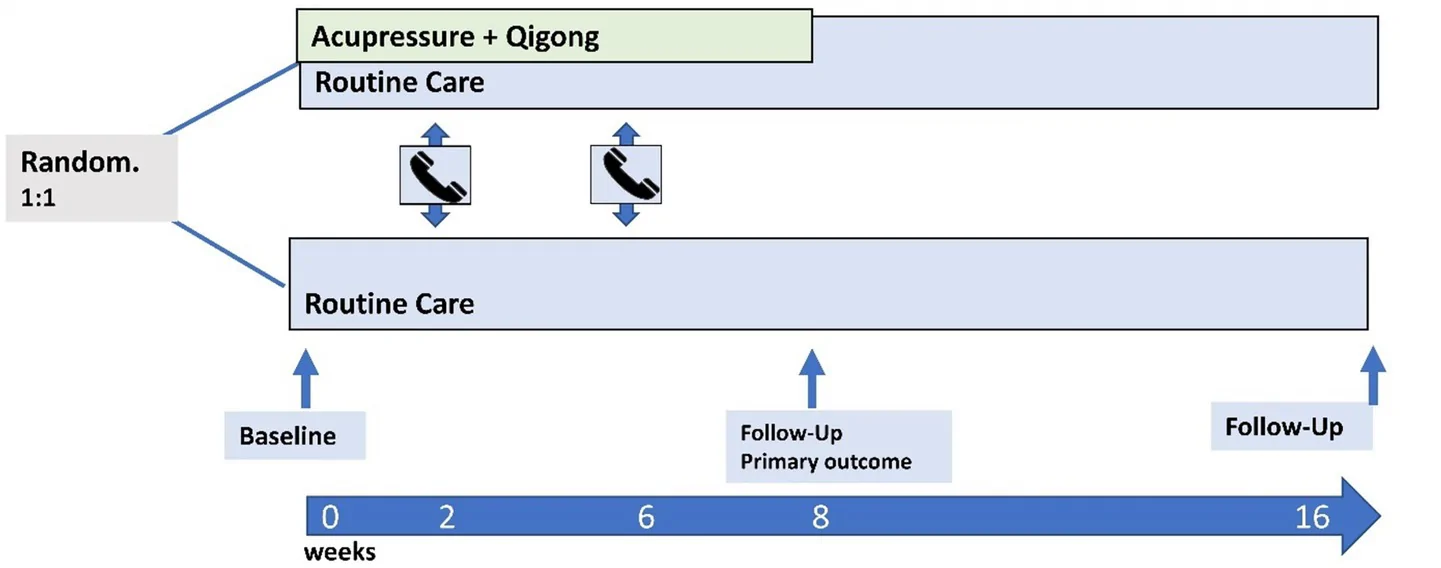
Timeline of the ACUQiG trial. All patients received calls in week 2 and week 6 to ensure adherence to diaries and questionnaires.

The ACUQiG-study was designed as an open-label, two-arm, randomized controlled trial conducted at a single center utilizing a mixed-methods approach including a qualitative study (methods and results of the qualitative study will be presented elsewhere; see Figure 1). Its primary aim was to evaluate the effects of an 8-week program combining self-applied acupressure and Qigong on outcomes related to PCS and fatigue in affected patients. This intervention was added to a self-care complementary medicine leaflet that was given to all participating patients and could be used as needed. Individual study participants were randomized at a 1:1 ratio into two treatment groups, using block randomization with variable block length. The randomization was performed via a computer-generated concealed randomized list, and allocation was revealed to the investigator after randomization of the individual patient [SAS for Windows (Version 9.4)]. A sub-study examined a randomly determined sample of the Berlin patients from both groups and assessed physical outcomes, such as handgrip strength, spirometry, or postural cardiac response; these examinations during the follow-ups were performed by a blinded assessor. The results will be published separately.

The trial was performed at the outpatient clinic for complementary and integrative medicine and prevention (CIM) at the Institute of Social Medicine, Epidemiology and Health Economics at the Charité – Universitätsmedizin Berlin, Germany. Information regarding the trial was published on websites of the universities PCS—network, self-help groups, and via posters in specialized centers for fatigue. Patient recruitment and assessments were conducted both at the outpatient clinic and through telemedicine across Germany. Patient recruitment started in June 2022 and ended in June 2023. The study received ethical approval from the ethics committee of the Charité – Universitätsmedizin Berlin (A2/073/22). All participants provided oral and written informed consent.

Several amendments were made to the study protocol after its initial design. The sleep quality PSQI was added at the start of the trial. Additionally, the follow-up phone calls were reduced from three to two, as patients had fewer questions than anticipated. The cohort size was increased by 35 participants to facilitate subgroup analyses, and a late follow-up assessment in week 104 was introduced.

All participants were provided with diaries to document their use of strategies outlined in the CIM self-care leaflet. Intervention group participants additionally logged their daily acupressure practice and weekly Qigong sessions. Telephone follow-ups were conducted in weeks 2 and 6 to offer guidance and address any adverse events.

All data from the electronic case report forms and online questionnaires (from the baseline examination and the questionnaires at baseline, week 8, and week 16) and the paper diaries (kept at home by the patients and sent via mail) were recorded in an online database using pseudonyms or entered directly there by the patients via an individualized study link. The current data protection regulations were applied.

### 2.2 Eligibility criteria and participants

Inclusion criteria were based on the WHO criteria for post-COVID. Included were adults aged 18–60 years who experienced chronic fatigue following a confirmed SARS-CoV-2 infection verified by PCR testing or detection of SARS-CoV-2 antibodies and consecutive persisting symptoms starting at least 12 weeks before enrollment in the trial.

At inclusion, patients were required to present with at least three of the following seven symptoms, assessed during a visit in the outpatient clinic or a video consultation with a physician: sleep disturbances, headaches, joint/muscle pain, anxiety/depression, memory/concentration issues, post-exertional malaise (PEM), or altered/diminished sense of smell (dysosmia/anosmia). Physical capacity had to measure no more than 60 mm on a Visual Analog Scale (VAS; 0–100 mm, with 0 indicating no capacity and 100 representing full capacity). Additionally, participants had to have 65 points or less on the physical function subscale of the Short-Form-36 (SF-36 PFS), a measure of limitations in daily physical activities with lower scores indicating greater impairment. The SF-36 PFS is commonly used as a primary outcome measure in studies of patients with post-infectious diseases characterized by exertion intolerance. Access to the necessary technology for online participation was also required.

The following exclusion criteria were defined: fatigue predating the SARS-CoV-2 infection, severe PEM alternative conditions capable of causing chronic fatigue (e.g., major depression, cancer, multiple sclerosis, fibromyalgia, or substance abuse), severe coexisting illnesses (e.g., significant pulmonary, cardiac, psychiatric, or infectious conditions) that might impact participation or outcomes, opioid use within the week before enrollment or chronic recreational drug use during the study, planned start or discontinuation of psychotherapy during the trial, pregnancy or breastfeeding, participation in another interventional clinical trial, ongoing or planned disability pension applications, and planned rehabilitation for PCS during the study period.

To assess the severity of post-exertional malaise (PEM), we used the DePaul Symptom Questionnaire–Post-Exertional Malaise (DSQ-PEM), which assesses severity, frequency, and duration of PEM and differentiates between PEM triggered by minor daily activities, whereas severe PEM is defined as symptom exacerbation following minimal activities. To facilitate assessment, we gave verbal examples of these activity levels: minor activities include household chores such as emptying the dishwasher or vacuuming, while minimal activities include basic self-care tasks such as brushing one’s teeth or changing clothes.

### 2.3 Study interventions

**Figure 2:**
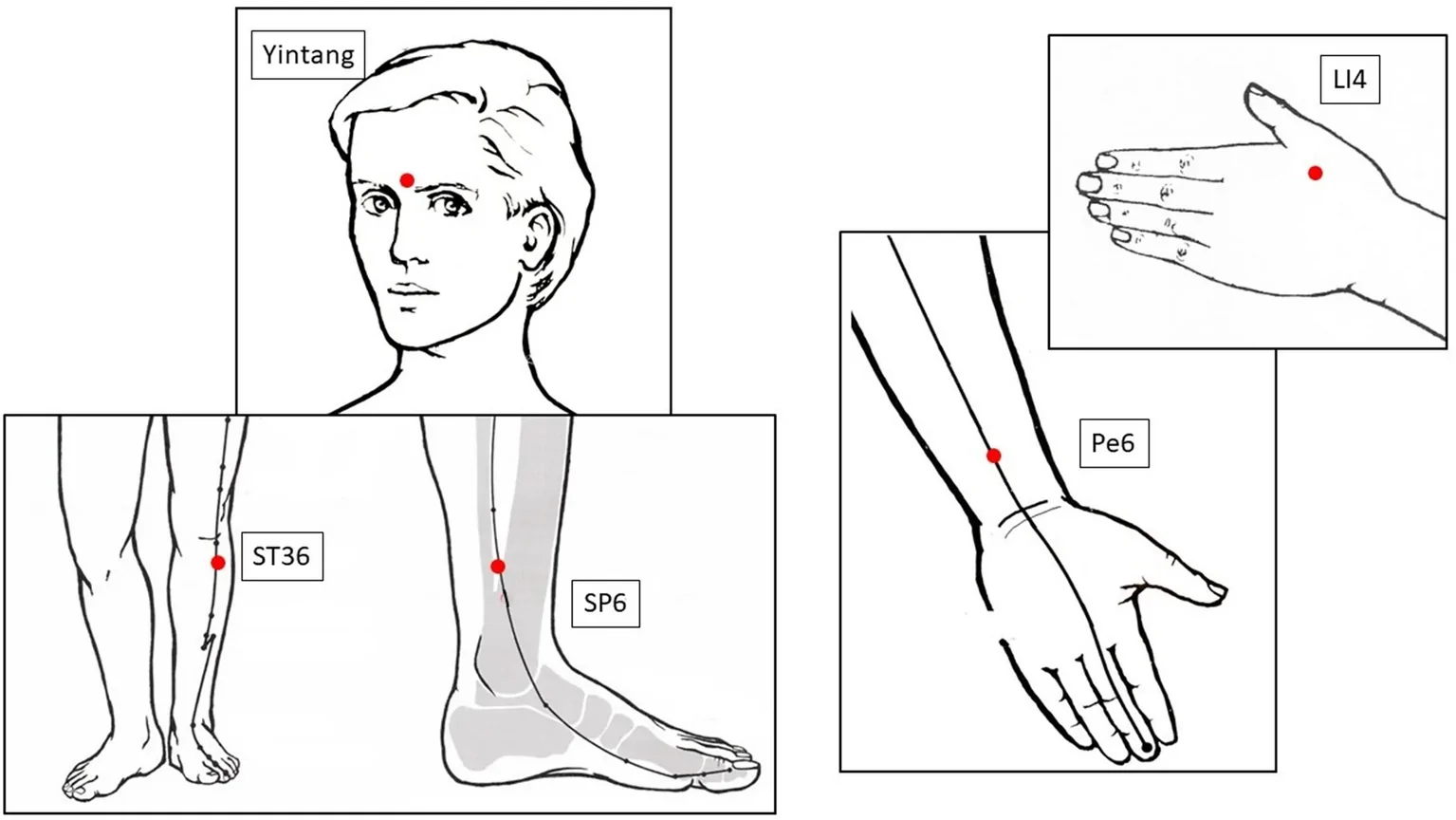
Obligatory acupressure points. WHO nomenclature for acupuncture points: LI4: large intestine 4, ST36: stomach 36, SP6: spleen 6, Pe6: pericardium 6. Figure modified with the permission of Stux, G. Lehrbuch und Atlas der Akupunktur, Springer Verlag 1985.

The acupressure and Qigong program was collaboratively developed with experts from the International Society for Chinese Medicine (SMS), Munich, Germany, and informed by previous research (14, 15). Patients in the intervention group received training in self-applied acupressure from an experienced study physician trained in acupuncture and related techniques from TCM, and patients were provided with instructional materials including a video tutorial and a printed manual. The semi-standardized acupressure protocol consisted of nine mandatory points and up to two additional symptom-specific points, as chosen by the participants. The mandatory points included Yintang and the bilateral points Large Intestine 4, Pericardium 6, Stomach 36, and Spleen 6 (see Figure 2). Patients could optionally select up to two additional points based on their PCS symptoms: Anmian, Liver 3, Gallbladder 20, Gallbladder 34, Governor Vessel 23/24, or Lung 7. Participants massaged these points daily for approximately 2 min per point, totaling at least 18 min daily for 8 weeks. Participants experiencing pain or bruising at an acupressure point were advised to reduce pressure or avoid the area until discomfort subsided. Acupressure duration was informed by prior acupressure studies on fatigue with the aim to balance efficacy and adherence (15).

In addition, a twice-weekly online Qigong class (45 min each) was offered as a live group session using the Zoom program. All Qigong therapists had completed a Qigong training program meeting the requirements of the German Central Prevention Examination Board—300 to 320 h over a minimum of 24 months, with a focus on Traditional Chinese Medicine (TCM). The Qigong program was offered for two different degrees of severity, basic and intermediate exercise sets, which could be selected by patients according to their symptoms. Initially, participants performed simple exercises, often in seated or lying positions, derived from the Crane exercises, tonifying spleen/stomach and kidney energy as well as strengthening lung and heart energy, and gentle stretching. After 4 weeks, intermediate exercises could be used if possible, incorporating more advanced techniques, breathing exercises, and vocalizations (“hu” and “xie”) from the six healing sounds (19). Patients paced themselves, pausing as needed based on perceived exertion. For those finding Qigong exercises too strenuous, modifications were encouraged, such as performing the exercises seated or reclining.

Both groups received a CIM-leaflet; the control group received only the CIM-leaflet, and in the first 8 weeks, the same number of phone calls. Incentive for participation was the offer to receive an acupressure manual and videos of the recorded Qigong sessions. All participants could continue symptomatic treatments such as NSAIDs, sleep medication, antidepressants, over-the-counter medications, herbal remedies, prescribed physical or occupational therapy, and practices like yoga or meditation, which were recorded in the diaries. However, additional acupuncture, acupressure, or Qigong therapies were prohibited during the study period. Both groups received written advice for self-care measures from an informational leaflet on post-viral symptoms and CIM self-care measures, with self-care guidance covering diet, herbs, supplements (including Western and Ayurvedic phytotherapy and traditional Chinese medicine dietary advice), hydrotherapy, breathing exercises, relaxation, and mind–body practices (20). Participants could select and experiment with different strategies. The aim of handing out this written advice was to provide all patients with possible but non-scientific strategies to potentially reduce their symptoms and to improve study recruitment and adherence.

### 2.4 Study outcomes

The primary outcome was the self-reported impairment of physical function after 8 weeks, assessed with the patient-reported physical function subscale of the SF-36, with higher values indicating better physical health (range 0–100). The SF-36 PFS is a most commonly used functional endpoint in fatigue research. Although the minimal clinically important difference (MCID) has been reported as 8 in kids, a more conservative threshold of 10 was used in this trial to define a secondary responder outcome assessed at 8 weeks (21). We acknowledge that a validated MCID for the SF-36 PFS in adults with PCS has not been established; we therefore selected a conservative threshold based on pediatric CFS/ME literature, which may not directly translate.

All secondary outcomes were collected at weeks 8 and 16. Secondary outcomes included the physical capacity (measured by a 100 mm VAS, with higher scores indicating better capacity) and headache severity [measured by VAS, with lower values indicating less pain) MCID for the VAS was >13 mm difference (22, 23)]; the Chalder Fatigue Questionnaire [CFQ, where lower scores signify better physical and mental health, MCID 2.3–3.3 points (24, 25)]; severity of post-exertional malaise (DSQ-PEM, with lower scores representing less malaise, a MCID has not yet been established); sleep quality [Pittsburgh Sleep Quality Index, PSQI, where lower scores indicate better sleep, a MCID of 3.0 has been suggested (26)]; health-related quality of life (EQ-5D-5L, where higher scores represent better quality of life); depressive symptoms [Patient Health Questionnaire-9, PHQ-9, MCID 14 points (27)]. Further assessments were expectation of efficacy at baseline, adherence, and patient global impression of change (PGIC) in week 8.

All adverse reactions and adverse events were recorded in patient diaries from weeks 1 to 8 and in electronic case report forms. In case of severe adverse events (SAEs), patients were instructed to contact the study center immediately via the contact info given at trial start. SAEs had to be reported to the principal investigator within less than 24 h.

### 2.5 Statistics

The sample size calculation was based on an anticipated minimal clinically important difference (MCID) of 10 points in the SF-36 PFS between treatment groups, with an expected standard deviation (SD) of 23 points (28). To achieve 80% power with a significance level of 5% in a two-sided *t*-test, 85 patients per group were required. To account for a projected 15% dropout rate, 100 participants per group were planned. A later protocol amendment allowed for the inclusion of up to 50 additional participants to enable subgroup analyses. Descriptive analysis included absolute and relative frequencies (percentages) for categorical variables, mean values, SD, median, range, and 95% confidence intervals for all continuous variables. For the analysis of the primary outcome, severity of the impairment of physical function measured by SF-36 PFS at week 8, the treatment groups (intervention/control) were compared using analysis of covariance (ANCOVA) with the baseline value of the SF-36 PFS as the covariate. Adjusted means are presented with two-sided 95% confidence intervals (CI) for each treatment group and the *p*-value of the group comparison (significance level of 5%, two-sided). All further analyses are considered exploratory (without adjustment for multiple testing). Secondary outcomes were analyzed with ANCOVA or logistic regression (depending on the scale of the outcome). Regression models included the factor treatment group and the respective baseline value (if available) as covariate. A repeated measures ANCOVA was performed for the SF-36 PFS at week 8 and 16 with the additional fixed factors time and time*treatment, and random factor “subject” in the model. Subgroup analyses were performed for the baseline variables PCS duration (</≥6 months), number of PCS symptoms (≤/>5), sex (female/male), menopause (yes/no, only in women), PEM (more than vs. max. 14 h of rest necessary to recover from minor household tasks), supplement use (yes/never), PCS medication use (yes/never) and depression (PHQ-9, ≤/>10). As a *post hoc* exploratory analysis, changes in SF-36 PFS and CFS were examined descriptively in both study groups, stratified by self-reported use of Ashwagandha (a fortifying plant used in fatigue in Ayurvedic medicine) during the first 8 weeks of the intervention. The statistical analyses were performed with R, version 4.4.1 (29). Following the intention-to-treat (ITT) principle, patients were analyzed according to the treatment to which they were randomized (without imputation of missing values, full analysis set). Analyses were conducted on complete cases. One per-protocol set was composed of participants who completed at least 60% of the acupressure treatments and 60% of the Qigong online sessions, who did not undergo plasmapheresis during the intervention weeks (PPP1). Another per-protocol set was similarly defined but did not exclude patients with plasmapheresis (PPP2). Additionally, the type of routine care therapies regarding PCS symptoms, including self-care measures from the CIM self-care leaflet, were analyzed descriptively from the diaries.

Data for safety, including adverse events and serious adverse events, were analyzed descriptively with the safety population, defined as receiving at least one acupressure or one Qigong treatment and having at least one post-baseline safety assessment.

## 3 Results

**Figure 3:**
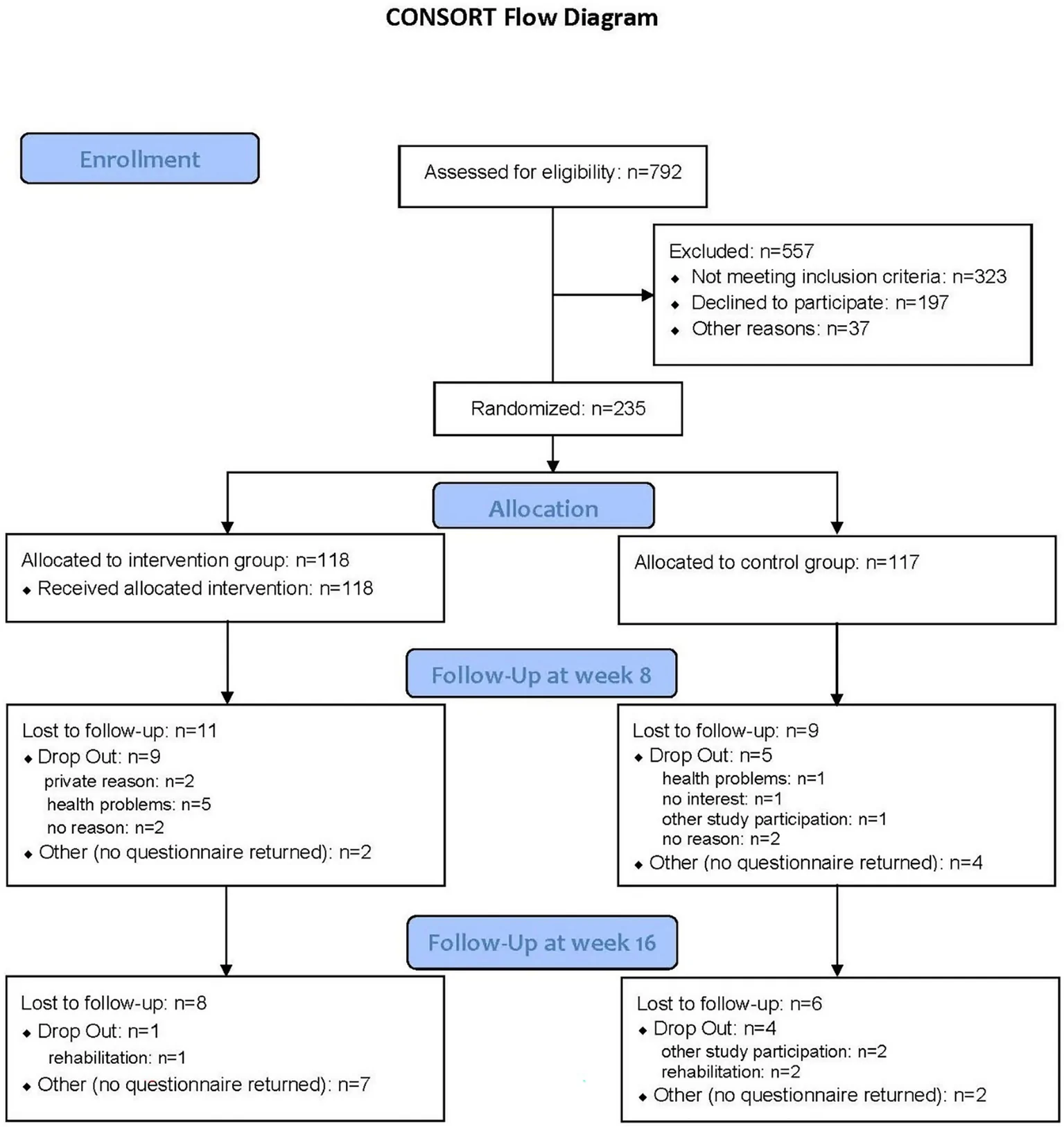
CONSORT flow diagram.

Between June 2022 and July 2023, 792 patients were screened for eligibility. Of these, 235 met the inclusion criteria, were randomized, and completed the baseline assessments (for CONSORT flow chart see Figure 3). Study participants were predominantly female individuals, but there were more male participants in the control group [22 (18.8%) vs. 11 (9.3%)]; otherwise, groups were comparable (see Table 1). Most participants were otherwise healthy, middle-aged individuals, more than half of them holding a university degree. More than 70% were on sick leave due to PCS, and their index infection had occurred, on average, more than a year (median 48 weeks) prior to trial participation (Table 2). Additionally, 82% had previous experience with CIM. Over 80% of participants experienced post-exertional malaise, characterized by recovery periods exceeding 14 h after minor activities; the mean number of additional symptoms apart from the fatigue was eight (see Table 2). Detailed pre-study self-medication and baseline off-label drug use have been published elsewhere (30).

**Table 1:** Patient characteristics at baseline.

Characteristic	Overall *N* = 235 *n* (%)/mean (SD)	Intervention *N* = 118 *n* (%)/mean (SD)	Control *N* = 117 *n* (%)/mean (SD)
Sex			
Female	200 (85.1%)	106 (89.8%)	94 (80.3%)
Male	33 (14.0%)	11 (9.3%)	22 (18.8%)
Diverse	2 (0.9%)	1 (0.8%)	1 (0.9%)
Age	42.1 (10.3)	42.6 (10.0)	41.7 (10.7)
Highest education			
University degree	157 (66.8%)	75 (63.6%)	82 (70.1%)
Vocational training	64 (27.2%)	38 (32.2%)	26 (22.2%)
College	13 (5.5%)	4 (3.4%)	9 (7.7%)
High school	1 (0.4%)	1 (0.8%)	0 (0.0%)
Occupation			
Sick leave	174 (74.0%)	84 (71.2%)	90 (76.9%)
Working full or part-time	47 (20.0%)	26 (22.0%)	21 (17.9%)
In training	4 (1.7%)	3 (2.5%)	1 (0.9%)
Pension/disability pension	3 (1.3%)	2 (1.7%)	1 (0.9%)
Looking for work	2 (0.9%)	0 (0.0%)	2 (1.7%)
Other	5 (2.1%)	3 (2.5%)	2 (1.7%)
BMI (kg/m^2^)	24.0 (4.7)	24.3 (4.5)	23.7 (4.9)
Menopause (of *n* = 200 women)	56 (28.0%)	31 (29.2%)	25 (26.6%)
Comorbidities			
Asthma	21 (8.9%)	11 (9.3%)	10 (8.5%)
Hypertension	16 (6.8%)	11 (9.3%)	5 (4.3%)
Depression/anxiety	15 (6.4%)	8 (6.8%)	7 (6.0%)
Hypercholesterinemia	4 (1.7%)	2 (1.7%)	2 (1.7%)
Chronic obstructive pulmonary disorder	1 (0.4%)	1 (0.8%)	0 (0.0%)
Smoking	25 (10.6%)	15 (12.7%)	10 (8.5%)
Recreational drug use	4 (1.7%)	1 (0.8%)	3 (2.6%)
Expectation (Qigong and acupressure)			
No improvement	1 (0.4%)	1 (0.8%)	0 (0.0%)
Some improvement	164 (69.8%)	77 (65.3%)	87 (74.4%)
Strong improvement	70 (29.8%)	40 (33.9%)	30 (25.6%)
Use of complementary medicine in the past	192 (81.7%)	98 (83.1%)	94 (80.3%)

**Table 2:** Baseline characteristics regarding index infection and actual PCS symptoms.

Characteristic	Overall *N* = 235 *n* (%)/mean (SD)	Intervention *N* = 118 *n* (%)/mean (SD)	Controls *N* = 117 *n* (%)/mean (SD)
Time since index infection [weeks]	56.4 (34.2)	56.2 (34.8)	56.7 (33.8)
Inpatient treatment due to acute SARS-CoV-2	10 (4.3%)	7 (5.9%)	3 (2.6%)
Number of vaccinations * before index infection			
0	74 (31.5%)	41 (34.7%)	33 (28.2%)
1	5 (2.1%)	2 (1.7%)	3 (2.6%)
2	41 (17.4%)	17 (14.4%)	24 (20.5%)
3	109 (46.4%)	53 (44.9%)	56 (47.9%)
4	6 (2.6%)	5 (4.2%)	1 (0.9%)
PCS related symptom scores			
Physical function (SF-36 PFS 0–100) §	42.8 (16.6)	43.2 (16.6)	42.4 (16.6)
Chalder Fatigue Scale (CFS 0–33) &	25.5 (4.3)	25.5 (4.3)	25.4 (4.3)
Physical function (VAS full capacity, 0–100) §	34.9 (12.4)	36.0 (12.5)	33.8 (12.2)
Post-exertional malaise (DSQ-PEM, 0–50) &	21.5 (8.7)	21.1 (8.7)	21.8 (8.7)
Quality of life (EQ-5D-5L index 0–1) §	0.6 (0.2)	0.6 (0.2)	0.6 (0.2)
Quality of life (EQ-5D-5L VAS 0–100) §	36.2 (15.1)	37.4 (16.3)	34.9 (13.7)
Depressiveness [PHQ-9 total score (0–27)] &	11.0 (4.1)	11.2 (4.5)	10.8 (3.7)
Headache (VAS 0–100) &	35.7 (25.6)	36.5 (27.0)	34.9 (24.1)
Sleep quality (PSQI 0–21) &	10.1 (3.7)	10.2 (3.7)	10.0 (3.6)
Additional PCS-related symptoms besides fatigue			
Intolerance to physical activity	230 (97.9%)	114 (96.6%)	116 (99.1%)
Fluctuation of symptoms	225 (95.7%)	115 (97.5%)	110 (94.0%)
Memory/concentration impairment	219 (93.2%)	110 (93.2%)	109 (93.2%)
Joint/muscle pain	200 (85.1%)	102 (86.4%)	98 (83.8%)
Sleep disturbance	183 (77.9%)	96 (81.4%)	87 (74.4%)
Headache	172 (73.2%)	85 (72.0%)	87 (74.4%)
Post-exertional malaise	155 (66.0%)	71 (60.2%)	84 (71.8%)
Dyspnea	155 (68.0%)	78 (68.4%)	77 (67.5%)
Vertigo	128 (54.5%)	63 (53.4%)	65 (55.6%)
Anxiety/depressiveness	110 (46.8%)	59 (50.0%)	51 (43.6%)
Spontaneous sweating	86 (36.6%)	50 (42.4%)	36 (30.8%)
Dysosmia/anosmia	53 (22.6%)	31 (26.3%)	22 (18.8%)
Number of symptoms (0 to 12)	8.2 (1.7)	8.3 (1.7)	8.1 (1.6)

§: higher values = better health, &: lower values = better health. *: vaccination date plus 14 days was calculated to assume sufficient immune response.

Patients were included in the study at the physician’s discretion in specific cases: one breastfeeding patient was allowed to participate as she was in the process of weaning her toddler off nighttime breastfeeding. Another patient was undergoing psychotherapy for neurocognitive training but did not have a diagnosed anxiety or depressive disorder. Four patients reported occasional use of cannabis or recreational drugs but not on a regular basis. Three participants could only provide antigen test or antibody evidence of SARS-CoV-2 infection, as PCR testing was unavailable during a temporary shortage in Germany. One participant unexpectedly began physical rehabilitation and was excluded at the start of the rehabilitation program. There was one case of erroneous inclusion: a participant with an SF-36 PFS score of 70 was included despite exceeding the cutoff.

### 3.1 Study intervention

To evaluate the choice and use of self-care measures recommended in the CIM self-care leaflet provided to both groups, we analyzed participants’ diary entries from the first 8 weeks. Both groups were mostly comparable in the number of different self-care measures used over the 8 weeks: according to the diaries, the intervention group used a mean of 6.3 different self-care measures per person of any kind from the CIM self-care leaflet (maximum 13 self-care measures), and the control group a mean of 6.6 different applications per person (Table 3). In the intervention group, adherence was good: Acupressure was practiced on average for 43 days (SD = 11) out of the 56-day intervention period (8 weeks), corresponding to 76.8% of the planned applications. Participation in Qigong was lower, with patients attending a mean of 10 sessions (SD = 4) out of the 16 planned sessions (62.5%).

**Table 3:** Frequencies of chosen self-care measures from the complementary and integrative medicine (CIM) self-care leaflet according to the diaries from week 1 through 8.

Self-care measure/total uses	Intervention N = 107 *n* (%)/mean (SD)	Control N = 110 *n* (%)/mean (SD)
Relaxation	89 (83%)	92 (84%)
Breathing exercises	85 (79%)	97 (88%)
Cold water pours	69 (64%)	78 (71%)
Lemon balm tea	53 (50%)	49 (45%)
Lavender aroma oil	49 (46%)	45 (41%)
Ashwagandha capsules	48 (45%)	76 (69%)
Arnica oil massage	48 (45%)	49 (45%)
Dry brushing of legs	41 (38%)	49 (45%)
Lavender tea	29 (27%)	33 (30%)
Ginger compresses	27 (25%)	38 (35%)
Oil pulling	26 (24%)	32 (29%)
Valerian	23 (21%)	25 (23%)
Inhalations	22 (21%)	32 (29%)
Gingko capsules	17 (16%)	29 (26%)
Frequency: mean (SD) per person	6.3 (2.8)	6.6 (3.1)

The proportion of missing data for the primary outcome was low and similar between groups (intervention: 9%, control: 8%). At week 8, the adjusted mean SF-36 PFS score was 55.4 (95% CI, 51.8–59.1) in the intervention group and 51.2 (47.6–54.9) in the control group. The adjusted between-group difference was 4.2 points (−0.9 to 9.4, *p* = 0.109). In the per-protocol analysis (PPP1, intervention: *n* = 54; control: *n* = 108), results were consistent with the primary analysis: the adjusted mean difference in SF-36 PFS at week 8 was 5.3 points (−1.3 to 11.8, *p* = 0.116). Analysis results using the PPP2 were similar. A repeated measures ANCOVA across weeks 8 and 16, adjusted for baseline SF-36 PFS, confirmed the findings of the primary analysis (Table 4). For the results of the descriptive analysis of outcomes, see Supplementary Table S1.

**Table 4:** Primary and secondary endpoints: results from analysis of covariance (ANCOVA) in the full analysis set.

Assessment tool	Intervention	Control	Difference	*p*
	Adj. Mean (95% CI)	Adj. Mean (95% CI)	Adj. Mean difference (95% CI)	
Physical function (SF-36 PFS), week 8 (primary endpoint) [0–100, §]	55.4 (51.8 to 59.1)	51.2 (47.6 to 54.9)	4.2 (−0.9 to 9.4)	0.109
Physical function (SF-36 PFS), week 16 [0–100, §]	57.8 (53.6 to 62.0)	54.0 (49.9 to 58.1)	3.8 (−2.1 to 9.7)	0.207
SF-36 PFS, weeks 8 and 16 (repeated measures ANCOVA)	56.5 (52.9 to 60.2)	52.6 (48.9 to 56.2)	−3.97 (−9.10 to 1.17)	0.180
Chalder Fatigue Scale, week 8 [0–33, &]	21.4 (20.2 to 22.6)	23.2 (22.0 to 24.3)	−1.8 (−3.5 to −0.1)	0.040
Chalder Fatigue Scale, week 16 [0–33, &]	20.3 (19.0 to 21.6)	22.3 (21.0 to 23.6)	−2.0 (−3.9 to −0.2)	0.028
Physical function (VAS full capacity), week 8 [0–100, §]	48.2 (45.2 to 51.3)	42.5 (39.5 to 45.6)	5.7 (1.3 to 10.0)	0.011
Physical function (VAS full capacity), week 16 [0–100, §]	49.8 (45.7 to 53.9)	45.6 (41.6 to 49.7)	4.1 (−1.7 to 9.9)	0.160
Post-exertional malaise (DSQ-PEM), week 8 [0–50, &]	23.8 (22.2 to 25.4)	24.4 (22.8 to 26.0)	−0.6 (−2.9 to 1.6)	0.578
Post-exertional malaise (DSQ-PEM), week 16 [0–50, &]	23.1 (21.2 to 25.1)	24.5 (22.6 to 26.4)	−1.4 (−4.1 to 1.4)	0.322
Quality of life (EQ-5D-5L index), week 8 [0–1, §]	0.66 (0.62 to 0.69)	0.62 (0.58 to 0.66)	0.03 (−0.02 to 0.09)	0.214
Quality of life (EQ-5D-5L index), week 16 [0–1, §]	0.66 (0.62 to 0.71)	0.61 (0.57 to 0.65)	0.05 (−0.01 to 0.11)	0.105
Quality of life (EQ-5D-5L-VAS), week 8 [0–100, §]	45.9 (43.0 to 48.9)	43.0 (40.1 to 46.0)	2.9 (−1.3 to 7.1)	0.173
Quality of life (EQ-5D-5L-VAS), week 16 [0–100, §]	49.1 (45.3 to 52.8)	43.4 (39.7 to 47.1)	5.6 (0.3 to 10.9)	0.037
Depression (PHQ-9), week 8 [0–27, &]	9.6 (8.9 to 10.2)	10.0 (9.3 to 10.7)	−0.5 (−1.4 to 0.5)	0.352
Depression (PHQ-9), week 16 [0–27, &]	9.2 (8.4 to 10.0)	9.9 (9.1 to 10.7)	−0.7 (−1.8 to 0.4)	0.207
Headache (VAS), week 8 [0–100, &]	29.7 (26.1 to 33.3)	29.9 (26.4 to 33.5)	−0.2 (−5.3 to 4.8)	0.935
Headache (VAS), week 16 [0–100, &]	31.1 (26.6 to 35.5)	33.0 (28.7 to 37.4)	−2.0 (−8.2 to 4.2)	0.531
Sleep quality (PSQI), week 8 [0–21, &]	9.3 (8.8 to 9.9)	9.5 (8.9 to 10.0)	−0.1 (−0.9 to 0.7)	0.748
Sleep quality (PSQI), week 16 [0–21, &]	9.0 (8.4 to 9.6)	9.5 (8.8 to 10.1)	−0.5 (−1.3 to 0.4)	0.323

Adjusted means from ANCOVA models, adjusted for baseline values. Repeated measures ANCOVA for SF-36 PFS includes outcomes at weeks 8 and 16 with the baseline value as a covariate. §: higher values = better health, &: lower values = better health.

A responder analysis was conducted based on an improvement of at least 10 points in the SF-36 Physical Functioning Scale (SF-36 PFS) from baseline to week 8. A higher proportion of responders was observed in the intervention group (58.9%) compared to the control group (46.3%), OR 1.69, 95% CI, 0.98 to 2.92, *p* = 0.058 (Supplementary Tables S2a, S2b). In our sample, responders differed from non-responders in several clinically relevant baseline characteristics. Responders were on average younger (mean age 40.3 vs. 44.4 years), more often not on sick leave (27.4% vs. 14.7%), and had a shorter duration since SARS-CoV-2 infection (mean 50.9 vs. 62.3 weeks). Additionally, they reported slightly better health-related quality of life (EQ-5D-5L index 0.7 vs. 0.6; VAS 40.1 vs. 33.0) and tended to have less severe post-exertional malaise. However, after adjustment for these covariates in a post-hoc analysis, results remained essentially unchanged.

In the *post hoc* analysis with stratification by the intake of Ashwagandha during the first 8 weeks, no relevant differences were observed, neither for SF-36 PFS nor for CFS (results not shown).

**Figure 4:**
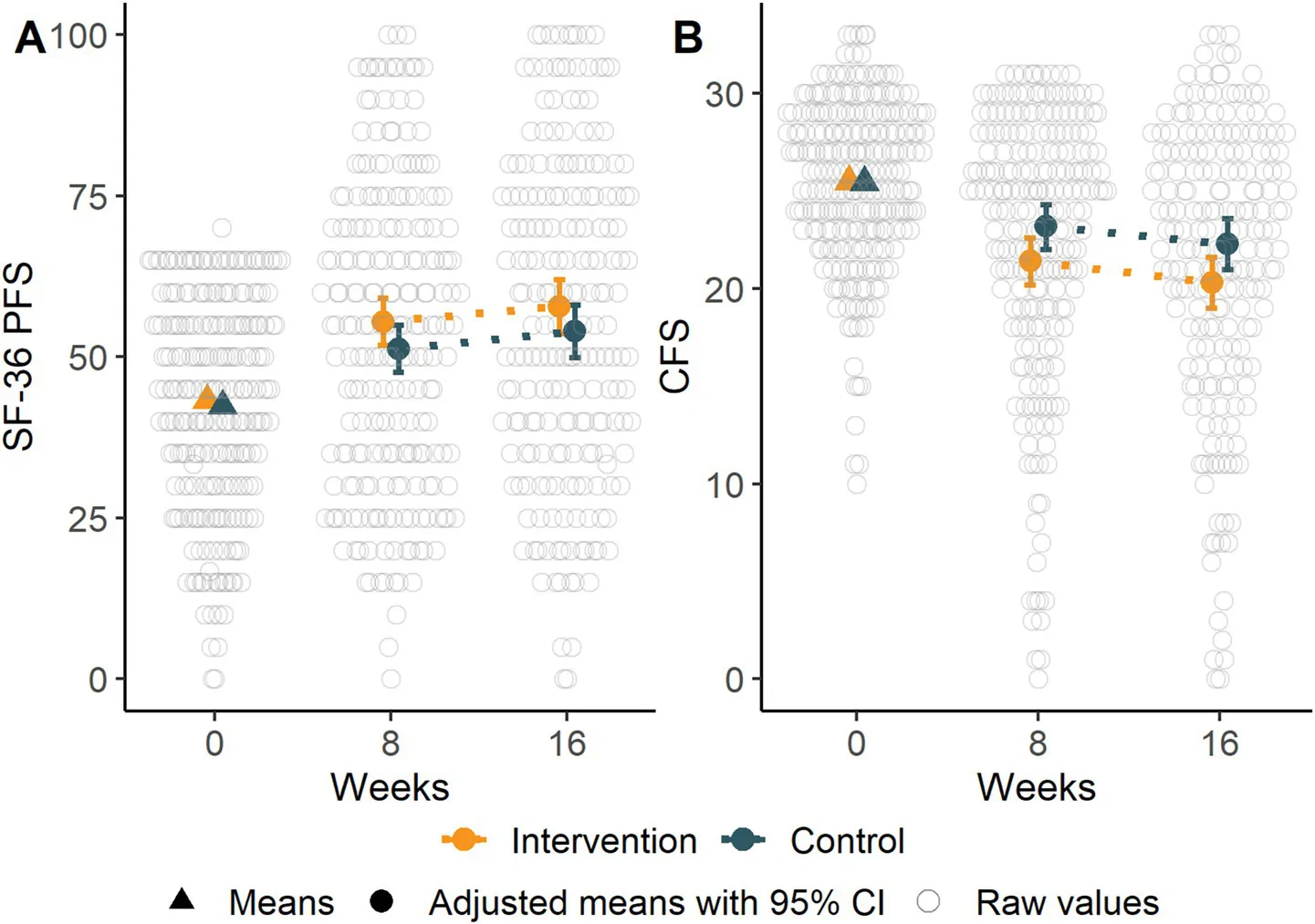
Distribution, means and adjusted means of **(A)** physical function (SF-36 PFS) and **(B)** Chalder Fatigue Scale (CFS).

Unadjusted values showed an improvement in physical functioning in the intervention group from baseline of 43.2 (SD 16.6) to 56.4 (24.2) in week 8 and to 58.7 (25.2) in week 16, while the improvement in the control group was a little smaller from a baseline of 42.4 (16.6) to 50.3 (23.0) in week 8 and 53.1 (24.9) in week 16 (see Supplementary Table S1). Regarding the secondary endpoints, the analysis showed minor differences in the CFS favoring the intervention group at weeks 8 and 16 (see Table 3, Figure 4), which failed to reach clinical relevance. These differences were not adjusted for multiple testing due to the exploratory nature of secondary analyses; therefore, they should be interpreted with caution and are not considered confirmatory.

In addition, the comparison of health-related quality of life (EQ-5D-5L) index values and VAS did not indicate a large difference between the groups. In addition, no substantial differences were observed between the groups for depression (PHQ-9), headache severity (VAS), or sleep quality (PSQI).

For results of exploratory subgroup analyses for physical function (SF-36), Chalder Fatigue Scale and sleep quality PSQI in relation to duration of PCS, age, supplement or medication use for PCS and the presence of PEM, see Supplementary Table S3.

The patient-reported global impression of change in week 8 was somewhat better in the intervention group. The median response in the intervention group was “a little bit better,” compared to “unchanged” in the control group. Furthermore, 23.8% of intervention group participants rated their improvement as “strong” or “very strong,” compared to 13.0% of control group participants.

Regarding safety, the combined study interventions can be considered safe. Acupressure leading to pain or bruising at the acupressure site was reported 12 times, while 42 times aggravation of preexisting symptoms was reported (such as PEM, headache, and dizziness). Qigong was linked to aggravation of preexisting symptoms 52 times. One severe adverse event, a recurrence of prolonged insomnia that led to hospitalization, was recorded in the intervention group but not linked to the intervention, and in the control group, 4 hospitalizations occurred: one due to severe headache, one case of tachycardia, one recurrence of myocarditis, and one prolonged vomitus, none of them related to the study procedures.

## 4 Discussion

Our trial findings indicate that participants who practiced acupressure and graded Qigong in addition to leaflet-based CIM self-care did not experience a relevant improvement in the physical function measured with the SF-36 PFS compared to an active control using only the leaflet-based CIM self-care. The responder analysis suggested a greater likelihood of clinically relevant improvement in SF-36 PFS scores within the intervention group compared to the control group, with less impaired individuals benefiting the most. Regarding the Chalder fatigue scale, no clinically relevant improvement was seen in the intervention group compared to the control group. The moderate adherence to Qigong (62.5%) may have diluted the treatment effect. However, our per-protocol analysis, though limited by small sample size, did not show a meaningful difference, suggesting that even in adherent patients, the intervention was not strongly effective. Although low level and graded, the Qigong sessions at defined appointments might have been too strenuous for some patients; the same with the acupressure routine, which might be demanding for patients experiencing intolerance for physical and mental activity. This is reflected in moderate adherence rates to Qigong and the adverse events, which mainly were aggravation of preexisting symptoms.

### 4.1 Strengths and limitations

Strengths of the study include its pragmatic design, which involves a self-care and online-based intervention aiming to support severely fatigued patients who cannot come to an outpatient clinic repetitively. Additionally, the study had a larger sample size compared to similar intervention studies on PCS and post-infective fatigue syndrome. The primary outcome measure, SF-36 PFS, is widely recognized as a valid and reliable tool for assessing interventions in this field. Objective measures of physical and social function, such as work attendance, were included in the study.

However, the study has some limitations. It was conducted at a single center, and blinding for group allocation was not possible due to the nature of the intervention, leaving the placebo effect uncontrolled. The study population comprised primarily nonhospitalized patients with moderate impairments, which may limit the generalizability of the results to individuals with severe sequelae following acute COVID-19. Our sample was predominantly female, highly educated, and moderately impaired, limiting generalizability to men, lower socioeconomic groups, and severely affected patients (e.g., those unable to participate in online sessions). Furthermore, current PCS definitions are broad and based solely on clinical characteristics; future research identifying subgroups may clarify which patients are most likely to benefit from this treatment, either alone or combined with therapies targeting specific pathophysiological mechanisms. The CIM self-care leaflet was provided to all patients as an incentive to participate. Although there was a potential risk of unequal application of self-care measures between groups, subsequent analysis demonstrated comparable use of adaptogens and other self-care practices, with no differences in the overall frequency of self-care activities between the two groups. The intervention group received more attention than the usual care group, which may have influenced outcomes, and the design cannot isolate the effect of the active intervention from the attention/support provided to the intervention group. Due to the open-label design and differential contact time between groups, we cannot rule out that the modest trends favoring the intervention group, particularly in the responder analysis and PGIC, are influenced by expectation bias or non-specific effects of increased attention. Nevertheless, most control group participants received medical care that could affect patient-reported outcomes, potentially mitigating the lack of a sham intervention in the control group.

### 4.2 Results in context

The SF-36 subscore for physical function SF-36 PFS has been widely used in trials on ME/CFS and is an established measure of impaired physical function (21, 31). In a representative sample of the healthy Norwegian population, the SF-36 PFS score for the healthy age group corresponding to our sample’s mean age was 90 (32), to put the severe baseline impairment of our SF-36 PFS of our cohort into perspective with a mean SF-36 PFS of 42.8 (SD 16.6).

A relevant comparison can be made with a Norwegian RCT involving 314 patients with mild to moderate PCS, where the SF-36 PFS was the primary outcome parameter (33). Participants underwent a cognitive behavioral therapy-based rehabilitation program tailored to individual needs, while controls received usual care. After 6 months of intervention, the intention-to-treat analysis showed a statistically and clinically significant improvement in SF-36 PFS, with a between-group score difference of 9.2 points (95% CI 4.3–14.2), *p* < 0.001, and Cohen’s *d* = 0.43. In comparison, our cohort had lower baseline SF-36 PFS scores, indicating greater impairment in physical functioning and a longer duration of PCS since the index infection. However, Chalder fatigue baseline scores were comparable to those in the trial by Nerli et al. In that study, the intervention group showed an improvement of −3.3 points (95% CI −5.1 to −1.6) compared to the control group.

Another study tested an online breathing and well-being program in 150 patients recovering from COVID-19 who continued to experience dyspnea—an issue commonly observed in our cohort as well. The English National Opera (ENO) Breath Program intervention consisted of 6 weeks of online breathing retraining using singing techniques and was compared to usual care (34). The primary outcome was the SF-36 at the end of the intervention. Compared to usual care, ENO Breathe was associated with an improvement in the mental health composite score (regression coefficient 2.42 [95% CI 0.03 to 4.80]; *p* = 0.047), but not in the physical composite score, which includes the SF-36-PFS (0.60 [95% CI −1.33 to 2.52]; *p* = 0.54). The improvement in both groups was approximately 4 points in the SF-36 PFS.

Currently, there is no clear consensus on what constitutes an MCID of the SF-36 PFS in individuals with PCS. A systematic review assessing the responsiveness of the SF-36 in randomized controlled trials of effective treatments found that none of the studies achieved improvements in physical health composite or mental health composite scores that exceeded the MCID threshold (35).

A cohort study on 70 U.S. COVID-positive patients examined symptom progression over 24 months. It found that post-COVID symptoms peaked, and quality of life reached its lowest point between 6- and 12-months post-infection. From this point, symptom prevalence declined, with 24% of participants experiencing PCS at 6–12 months, decreasing to 15% at 18 months and 6% at 24 months (36). Our planned extended follow-up at 24 months will provide further insight into whether our cohort follows a similar restitution pattern.

### 4.3 Implications for future research

The broad inclusion criteria may have masked differential treatment effects in specific PCS phenotypes. Our exploratory subgroup analyses, while hypothesis-generating only, suggest that future research should stratify by menopausal status and PCS duration. Future trial design could comprise an attention-matched sham control (e.g., sham acupressure + gentle stretching) to rule out expectation bias or non-specific effects of increased attention. While Qigong and acupressure are valuable complementary therapies, other holistic approaches, such as mindfulness-based stress reduction (MBSR) or Yoga, warrant investigation. The grade of post-exertional malaise could be an indicator of the tolerance of interventions. Future research should explore the synergistic effects of combining various complementary therapies with conventional treatments. Additionally, qualitative research examining patient experiences could provide valuable information about how these therapies influence perceived well-being and quality of life.

The use of online and remote delivery methods for therapies like Qigong and acupressure has shown promise, as seen as well in the ENO Breath Program (34). Future studies could explore the feasibility, acceptability, and efficacy of online platforms for delivering these interventions, particularly in populations who may have difficulty accessing in-person care due to physical limitations or geographical barriers.

## 5 Conclusion

The addition of acupressure and Qigong to a self-care advice leaflet did not provide a statistically significant or clinically relevant improvement in physical function in PCS patients with fatigue compared to the leaflet alone. However, given the high prevalence and significant burden of PCS, coupled with the current lack of approved treatments, this RCT contributes to assessing the effect of combined non-pharmacological therapies. Further high-quality clinical trials are needed to investigate the effects of multimodal self-care programs for managing PCS-related fatigue.

## Data Availability

The data manager, statistician, trial coordinator and principal investigator have access to the data. Data and instructional videos and manuals for the intervention can be obtained from the study center under reasonable request.
