# Supplementary material for: Acupressure and Qigong in post-infectious fatigue with impaired physical function: a randomized clinical trial
Source: Front Med (Lausanne). 2026 Aug 10;13:1872035. doi: 10.3389/fmed.2026.1872035 (PMC13500318; doi:10.3389/fmed.2026.1872035)
Supplement: Supplementary file 1 [file Data_Sheet_1.pdf]

Supplement:

Table S1: Descriptive comparison of outcomes between treatment groups across study time points (baseline, "pre"; after 8 weeks, "post intervention"; after 16 weeks, "follow up")

Assessment tool		Intervention	Control
		**N = 118 Mean (SD)**	**N = 117 Mean (SD)**
**SF36-PFS: Physical Function Scale (0-100)**	N		
pre	235	43.2 (16.6)	42.4 (16.6)
post intervention week 8	215	56.4 (24.2)	50.3 (23.0)
follow up week 16	201	58.7 (25.2)	53.1 (24.9)
**Chalder Fatigue Scale (0-33)**			
pre	235	25.5 (4.3)	25.4 (4.3)
post intervention week 8	214	21.4 (7.5)	23.2 (6.4)
follow up week 16	200	20.2 (7.5)	22.4 (7.1)
**Physical function (VAS full capacity) (0-100)**			
pre	235	36.0 (12.5)	33.8 (12.2)
post intervention week 8	215	49.2 (18.5)	41.6 (19.2)
follow up week 16		50.7 (23.6)	44.7 (21.9)
**EQ5D-Index**			
pre	235	0.6 (0.2)	0.6 (0.2)
post intervention week 8	214	0.7 (0.2)	0.6 (0.2)
follow up week 16	200	0.7 (0.2)	0.6 (0.3)
**EQ5D-VAS Health (0-100)**			
pre	235	37.4 (16.3)	34.9 (13.7)
post intervention week 8	214	47.2 (19.6)	41.8 (17.2)
follow up week 16		50.0 (22.4)	42.5 (20.2)
**PHQ9 (0-27)**			
pre	235	11.2 (4.5)	10.8 (3.7)
post intervention week 8	214	9.6 (4.8)	10.0 (4.5)
follow up week 16	200	9.2 (5.0)	9.8 (4.9)
**VAS headache (0-100)**			
pre	235	36.5 (27.0)	34.9 (24.1)
post intervention week 8	215	30.4 (25.1)	29.2 (22.0)
follow up week 16	200	31.8 (26.8)	32.3 (24.6)
**PSQI (0-21)**			
pre	230	10.2 (3.7)	10.0 (3.6)
post intervention week 8	213	9.3 (4.1)	9.5 (3.4)
follow up week 16	200	9.0 (3.9)	9.5 (4.2)
**Proportion on sick leave**			
pre	235	84 (71.2%)	90 (76.9%)
week 8	203	54 (76%)	63 (80%)
week 16	197	45 (68%)	58 (75%)

SF-36 -PFS: Short-Form-36 quality of life- -physical function subscale: higher values indicate less fatigue (the norm being >75 in this age group), MCID=10 points; Chalder Fatigue Scale: lower values indicate less fatigue, MCID= 3 points;

VAS100mm (visual analogue scale) -physical capacity: higher values indicate more capacity; PEM: post- exertional malaise. PHQ-9- Patient Health Questionnaire: lower values indicate less depressive symptoms; PSQI- Pittsburgh Sleep Inventory Quality Index: lower values indicate better sleep quality; EQ5D-Index (European Quality of Life 5 Dimensions): higher values indicate better quality of life, EQ5D-VAS: higher values indicate better quality of life.

Table S2a: Responder analysis with responder defined as improvement by at least 10 points on the SF-36 -PFS. Results from logistic regression adjusted for baseline SF-36 -PFS.

	PFS Responder<br>n (%)	Odds ratio (95% CI)	p-value
Intervention	63 (58.9%)	1.69 (0.98 - 2.92)	0.058
Controls	50 (46.3%)	Ref.	-

Table S2b: Responder vs non-responder baseline- characteristics

Characteristic	Overall<br>**N=235**<br>n (%) / mean (SD)	**Non-responder (n=102)**<br>n (%) / mean (SD)	**Responder (n=113)**<br>n (%) / mean (SD)
**Sex**			
Female	200 (85.1%)	86 (84.3%)	95 (84.1%)
Male	33 (14.0%)	14 (13.7%)	18 (15.9%)
Diverse	2 (0.9%)	2 (2.0%)	0 (0.0%)
**Age**	42.1 (10.3)	44.4 (9.9)	40.3 (10.3)
**University degree**	157 (66.8%)	68 (66.7%)	76 (67.3%)
**Sick- leave**	174 (74.0%)	86 (84.3%)	71 (62.8%)
**BMI (kg/m²)**	24.0 (4.7)	24.4 (5.3)	23.4 (3.5)
**Menopause** (of n=200 women)	56 (28.0%)	31 (36.0%)	19 (20.0%)
**Expectation of improvement (Qigong and acupressure)**			
Strong improvement	70 (29.8%)	29 (28.4%)	36 (31.9%)
**Use of complementary medicine in the past**	192 (81.7%)	85 (83.3%)	89 (78.8%)
**Time since index infection [weeks]**	56.4 (34.2)	62.3 (33.1)	50.9 (34.1)
**PCS related symptom scores**			
Physical function (SF-36 - PFS 0-100) §	42.8 (16.6)	43.7 (16.3)	42.2 (16.6)
Chalder Fatigue Scale (CFS 0-33) &	25.5 (4.3)	26.3 (3.9)	24.6 (4.4)
Physical function (VAS full capacity, 0-100) §	34.9 (12.4)	34.2 (12.1)	35.7 (12.9)
Post-exertional malaise (DSQ-PEM, 0-50) &	21.5 (8.7)	23.6 (8.5)	19.4 (8.7)
Quality of life (EQ-5D-5L index 0-1) §	0.6 (0.2)	0.6 (0.2)	0.7 (0.2)
Quality of life (EQ-5D-5L VAS 0-100) §	36.2 (15.1)	33.0 (13.1)	40.1 (16.1)
Depressiveness (PHQ-9 total score (0-27) &	11.0 (4.1)	11.5 (3.7)	10.7 (4.6)
Headache (VAS 0-100) &	35.7 (25.6)	36.8 (26.3)	36.3 (25.2)
Sleep quality (PSQI 0-21) &	10.1 (3.7)	10.4 (3.6)	9.8 (3.7)

Table S3: Exploratory subgroup analyses for physical function (SF-36), Chalder Fatigue Scale and sleep quality (PSQI).

Outcome	Subgroup	Intervention group	Control group	Difference (Intervention – control)
		Adj. Mean (95% CI)	Adj. Mean (95% CI)	Adj. Mean (95% CI)
Physical function (SF-36) at week 8	PCS duration < 6 months	56.4 (48.7 - 64.2)	56.9 (48.8 - 65.0)	-0.5 (-11.8 - 10.8)
	PCS duration ≥ 6 months	55.1 (51.0 - 59.3)	49.8 (45.7 - 53.9)	5.4 (-0.4 - 11.2)
Chalder Fatigue Scale at week 8	PCS duration < 6 months	20.3 (17.7 - 22.8)	23.5 (20.9 - 26.1)	-3.2 (-6.9 - 0.4)
	PCS duration ≥ 6 months	21.7 (20.4 - 23.1)	23.1 (21.7 - 24.4)	-1.4 (-3.3 - 0.6)
Sleep quality (PSQI) at week 8	PCS duration < 6 months	8.4 (7.2 - 9.6)	9.6 (8.3 - 10.8)	-1.2 (-2.9 - 0.5)
	PCS duration ≥ 6 months	9.6 (9.0 - 10.3)	9.5 (8.8 - 10.1)	0.2 (-0.7 - 1.1)
Physical function (SF-36) at week 8	#PCS symptoms ≤ 5	69.4 (60.5 - 78.3)	59.0 (51.0 - 67.1)	11.1 (-12.3 - 34.5)
	#PCS symptoms > 5	52.9 (49.0 - 56.7)	49.3 (45.3 - 53.2)	4.7 (-1.1 - 10.4)
Chalder Fatigue scale at week 8	#PCS symptoms ≤ 5	18.7 (15.7 - 21.7)	22.6 (19.9 - 25.3)	-2.0 (-9.6 - 5.6)
	#PCS symptoms > 5	21.9 (20.6 - 23.2)	23.3 (22.0 - 24.6)	-2.1 (-3.9 - -0.2)
Physical function (SF-36) at week 8	Female	55.3 (51.4 - 59.2)	50.0 (46.0 - 54.1)	5.2 (-0.4 - 10.9)
	Male	58.7 (47.3 - 70.1)	57.1 (48.8 - 65.4)	1.6 (-12.4 - 15.7)
Chalder Fatigue Scale at week 8	Female	21.7 (20.4 - 22.9)	23.7 (22.4 - 25.0)	-2.0 (-3.8 - -0.2)
	Male	18.3 (14.4 - 22.2)	20.9 (18.2 - 23.6)	-2.6 (-7.3 - 2.1)
Sleep quality (PSQI) at week 8	Female	9.6 (9.0 - 10.2)	9.7 (9.1 - 10.4)	-0.2 (-1.0 - 0.7)
	Male	7.4 (5.6 - 9.1)	8.5 (7.3 - 9.8)	-1.2 (-3.4 - 1.0)
Physical function (SF-36) at week 8	Not in menopause	54.7 (50.2 - 59.2)	51.7 (47.0 - 56.4)	3.0 (-3.6 - 9.5)
	In menopause	55.5 (48.4 - 62.7)	44.3 (36.5 - 52.1)	11.2 (0.7 - 21.8)
Chalder Fatigue Scale at week 8	Not in menopause	21.4 (20.0 - 22.8)	24.1 (22.6 - 25.6)	-2.7 (-4.7 - -0.6)
	In menopause	22.3 (20.0 - 24.6)	22.6 (20.1 - 25.0)	-0.3 (-3.6 - 3.1)
Sleep quality (PSQI) at week 8	Not in menopause	9.6 (8.9 - 10.3)	10.0 (9.3 - 10.7)	-0.4 (-1.4 - 0.6)
	In menopause	9.7 (8.6 - 10.8)	9.3 (8.1 - 10.5)	0.4 (-1.2 - 2.1)
Physical function (SF-36) at week 8	No PEM	56.6 (51.4 - 61.8)	55.4 (49.6 - 61.1)	1.2 (-6.5 - 9.0)
	PEM	54.2 (49.0 - 59.5)	48.5 (43.8 - 53.1)	5.8 (-1.3 - 12.8)
Chalder Fatigue Scale at week 8	No PEM	20.2 (18.5 - 21.8)	22.7 (20.8 - 24.6)	-2.5 (-5.0 - -0.0)
	PEM	22.7 (21.0 - 24.4)	23.5 (21.9 - 25.0)	-0.8 (-3.1 - 1.5)
Sleep quality (PSQI) at week 8	No PEM	8.6 (7.8 - 9.3)	9.5 (8.6 - 10.4)	-0.9 (-2.1 - 0.2)
	PEM	10.2 (9.4 - 11.0)	9.5 (8.8 - 10.2)	0.7 (-0.3 - 1.8)
Physical function (SF-36) at week 8	No supplements	54.6 (46.7 - 62.4)	54.8 (46.9 - 62.7)	-0.3 (-11.4 - 10.9)

	Supplement use	55.7 (51.6 - 59.8)	50.3 (46.2 - 54.4)	5.4 (-0.4 - 11.3)
Chalder Fatigue Scale at week 8	No supplements	22.3 (19.7 - 24.9)	22.3 (19.7 - 24.8)	-0.0 (-3.7 - 3.6)
	Supplement use	21.1 (19.8 - 22.5)	23.4 (22.1 - 24.7)	-2.2 (-4.2 - -0.3)
Sleep quality (PSQI) at week 8	No supplements	9.8 (8.6 - 11.0)	9.5 (8.2 - 10.7)	0.3 (-1.4 - 2.0)
	Supplement use	9.2 (8.6 - 9.9)	9.5 (8.9 - 10.1)	-0.3 (-1.1 - 0.6)
Physical function (SF-36) at week 8	No PCS medication	57.1 (52.5 - 61.8)	54.0 (49.4 - 58.5)	3.2 (-3.4 - 9.7)
	PCS medication	52.9 (47.1 - 58.7)	46.7 (40.8 - 52.6)	6.2 (-2.0 - 14.5)
Chalder Fatigue Scale at week 8	No PCS medication	21.0 (19.4 - 22.5)	22.4 (20.9 - 23.9)	-1.4 (-3.6 - 0.7)
	PCS medication	22.1 (20.1 - 24.0)	24.4 (22.5 - 26.3)	-2.3 (-5.0 - 0.4)
Sleep quality (PSQI) at week 8	No PCS medication	9.2 (8.5 - 9.9)	9.2 (8.5 - 9.9)	-0.0 (-1.0 - 1.0)
	PCS medication	9.5 (8.7 - 10.4)	9.9 (9.0 - 10.8)	-0.3 (-1.6 - 0.9)
Physical function (SF-36) at week 8	Low PHQ-9 *	60.2 (54.8 - 65.7)	55.3 (49.8 - 60.7)	4.9 (-2.7 - 12.6)
	High PHQ-9 *	51.8 (47.0 - 56.6)	48.0 (43.2 - 52.8)	3.8 (-3.0 - 10.6)
Chalder Fatigue Scale at week 8	Low PHQ-9 *	21.0 (19.2 - 22.8)	22.5 (20.7 - 24.4)	-1.6 (-4.1 - 1.0)
	High PHQ-9 *	21.7 (20.1 - 23.3)	23.6 (22.0 - 25.2)	-1.9 (-4.2 - 0.3)

*Scale for Depression PHQ9: low PHQ-9 ... PHQ-9 ≤ 10, high: >10
